# Inflammation Caused by Praziquantel Treatment Depends on the Location of the *Taenia solium* Cysticercus in Porcine Neurocysticercosis

**DOI:** 10.1371/journal.pntd.0004207

**Published:** 2015-12-11

**Authors:** Carla Cangalaya, Mirko Zimic, Miguel Marzal, Armando E. González, Cristina Guerra-Giraldez, Siddhartha Mahanty, Theodore E. Nash, Hector H. García, Cysticercosis Working Group in Peru

**Affiliations:** 1 Laboratorio de Inmunopatología en Neurocisticercosis, Facultad de Ciencias y Filosofía, Universidad Peruana Cayetano Heredia, Lima, Perú; 2 Laboratorio de Bioinformática y Biología Molecular, Facultad de Ciencias y Filosofía, Universidad Peruana Cayetano Heredia, Lima, Perú; 3 Departamento de Ciencias Celulares y Moleculares, Facultad de Ciencias y Filosofía, Universidad Peruana Cayetano Heredia, Lima, Perú; 4 Facultad de Medicina Veterinaria, Universidad Nacional Mayor de San Marcos, Lima, Perú; 5 Laboratory of Parasitic Diseases, National Institute of Allergy and Infectious Diseases, National Institutes of Health, Bethesda, Maryland, United States of America; 6 Unidad de Cisticercosis, Instituto Nacional de Ciencias Neurológicas, Lima, Perú; Fundaçao Oswaldo Cruz, BRAZIL

## Abstract

**Background:**

Neurocysticercosis (NCC), infection of the central nervous system by *Taenia solium* cysticerci, is a pleomorphic disease. Inflammation around cysticerci is the major cause of disease but is variably present. One factor modulating the inflammatory responses may be the location and characteristics of the brain tissue adjacent to cysticerci. We analyzed and compared the inflammatory responses to cysticerci located in the parenchyma to those in the meninges or cysticerci partially in contact with both the parenchyma and the meninges (corticomeningeal).

**Methodology/Principal Findings:**

Histological specimens of brain cysticerci (n = 196) from 11 pigs naturally infected with *Taenia solium* cysticerci were used. Four pigs were sacrificed after 2 days and four after 5 days of a single dose of praziquantel; 3 pigs did not receive treatment. All pigs were intravenously injected with Evans Blue to assess disruption of the blood-brain barrier. The degree of inflammation was estimated by use of a histological score (ISC) based on the extent of the inflammation in the pericystic areas as assessed in an image composed of several photomicrographs taken at 40X amplification. Parenchymal cysticerci provoked a significantly greater level of pericystic inflammation (higher ISC) after antiparasitic treatment compared to meningeal and corticomeningeal cysticerci. ISC of meningeal cysticerci was not significantly affected by treatment. In corticomeningeal cysticerci, the increase in ISC score was correlated to the extent of the cysticercus adjacent to the brain parenchyma. Disruption of the blood-brain barrier was associated with treatment only in parenchymal tissue.

**Significance:**

Inflammatory response to cysticerci located in the meninges was significantly decreased compared to parenchymal cysticerci. The suboptimal inflammatory response to cysticidal drugs may be the reason subarachnoid NCC is generally refractory to treatment compared to parenchymal NCC.

## Author Summary

The cystic larvae of the pork tapeworm *Taenia solium* may affect the human brain causing neurocysticercosis (NCC), a very frequent cause of neurological symptoms in developing countries. The clinical expression and response to treatment of human NCC are related to the location of cysticerci inside (intraparenchymal) or outside the brain parenchyma (extraparenchymal NCC). We used a naturally infected pig model to assess the characteristics of inflammation around brain cysticerci of parenchymal, meningeal and mixed locations. There were no major differences in inflammation without treatment. After antiparasitic treatment with praziquantel, inflammation around parenchymal brain cysticerci increased in comparison to meningeal located cysticerci. Cysticerci partially surrounded by both brain parenchyma and meninges showed increased inflammation in relation to the extent of the cysticercus in the brain parenchyma. The location of cysticerci within the brain is a factor that determines the extent and degree of the immune response following anticysticidal treatment. Similar changes may occur in treated human infections. Our work could contribute to explain the differences in response to antiparasitic treatment in different forms of human neurocysticercosis.

## Introduction

Neurocysticercosis (NCC), infection of the central nervous system (CNS) by the larval stage (cysticercus) of the parasitic helminth *Taenia solium*, is a common disease in regions where pigs are raised and allowed to roam freely [1,2]. NCC is a major cause of epileptic seizures in developing countries and therefore a serious public health problem [2]. Seizures and other symptoms of NCC depend on the number, location and distribution of cysticerci, as well as the degree of brain inflammation and developmental stage of the parasite, giving rise to a wide variety of manifestations [3,4].

The adult tapeworm, which resides in the small intestine of a human carrier, produces embryonated eggs containing embryos called oncospheres. After ingestion of contaminated feces by pigs or the accidental ingestion by humans, the oncospheres hatch, make their way to the bloodstream and mostly develop into cysticerci in the muscle, brain and subcutaneous tissues [2,5]. In the brain, the distribution of cysticerci generally follows the distribution of blood [3]. Cysticerci can be lodged in the parenchyma of the brain, cerebellum or brainstem, ventricles, subarachnoid space and spine [2,3]. According to cysticercal location there are two main forms of NCC: parenchymal and extraparenchymal disease (in which cysticerci have ventricular and/or subarachnoid locations) [2,3].

The clinical presentation, pathophysiology and treatment differ depending on the location and stage of cysticerci, degree of inflammation and other variables. Parenchymal NCC is associated with seizures or focal neurological signs. This form of NCC is relatively easy to treat, and has a fairly good prognosis compared to extraparenchymal NCC [6,7], which commonly causes hydrocephalus and intracranial pressure, and is associated with a poor prognosis [8,9].

Histology and medical imaging (computed tomography and magnetic resonance) have been useful to study and compare inflammation in human [10,11] and porcine NCC [12,13,14], including the effects of anthelmintic treatment [15] or cysticerci distribution [16,17]. However information of the association between anthelmintic treatment, inflammation and cysticercal location is scarce.

The vessels in parenchymal and meningeal tissue differ in the constitutive proteins of the junctional complex, susceptibility to changes in permeability of the blood-brain barrier (BBB), expression of cytokines that regulate BBB function and the type and number of associated astrocytes [18,19,20]. For the three highly vascularized tissue membranes of the meninges, dura mater, arachnoid and pia mater [21], the permeability of vessels in the pia mater is the most susceptible to perturbation and most critical in relation to the integrity of the BBB [22]. These tissue-specific characteristics could explain the differences on inflammation around the cysticercus in experimental murine models [19,20] and thus be involved in the severity of the inflammatory reaction elicited when the parasite degenerates–naturally or after anthelmintic therapy [2]. Cysticerci can be totally embedded within parenchymal or meningeal tissue or partially adjacent to both the brain parenchyma and meninges (corticomeningeal).

This study used a naturally infected pig as disease-based model to quantify and compare the inflammatory response following cysticidal treatment to cysticerci located in the brain parenchyma, meninges or both tissues.

## Materials and Methods

### Experimental design and treatment groups

This was a cross-sectional study. The methodology including pig husbandry, injection of Evans blue, use and dosing of pigs with praziquantel, euthanasia, as well as extraction, fixation and histopathological analysis of brain cysticerci is described in previously published reports employing the *T*. *solium* naturally infected pig model [23,27]. Brains from 11 pigs from Huancayo (Andean region endemic for NCC), naturally infected with *Taenia solium* **cysticerci** were used. The age of the pigs was between 2 and 4 years and the weight range was 42 kg to 107 kg. The animals had been randomly allocated to different groups: 3 pigs did not receive any drug (untreated, D0) and 8 received a single oral dose of 100 mg/kg praziquantel (Saniquantel 10%, Montana SA, Peru) which results in consistent cysticercal damage and subsequent enhanced host inflammatory responses [15,23,27]. Of these, 4 were sacrificed after two days (D2) and 4 after five days of the anthelmintic treatment (D5). All pigs were injected with Evans blue (EB, 80 mg/Kg; Sigma-Aldrich, St. Louis, MO) two hours before euthanasia [23].

### Ethical considerations

The primary study was performed at the Universidad Nacional Mayor de San Marcos, Lima, Peru, under the protocol “Evaluación de la permeabilidad vascular en cerebro y músculo de cerdos naturalmente infectados con *Taenia solium*”, with Dr. Armando Gonzalez as principal investigator. The study protocol was approved by the Animal Ethics and Wellbeing Committee of the University -CEBA (Constancia de autorización ética No. 006, November 2010) and comply with the National Institutes of Health/AALC guidelines [23].

### Selection of samples

**Fig 1:**
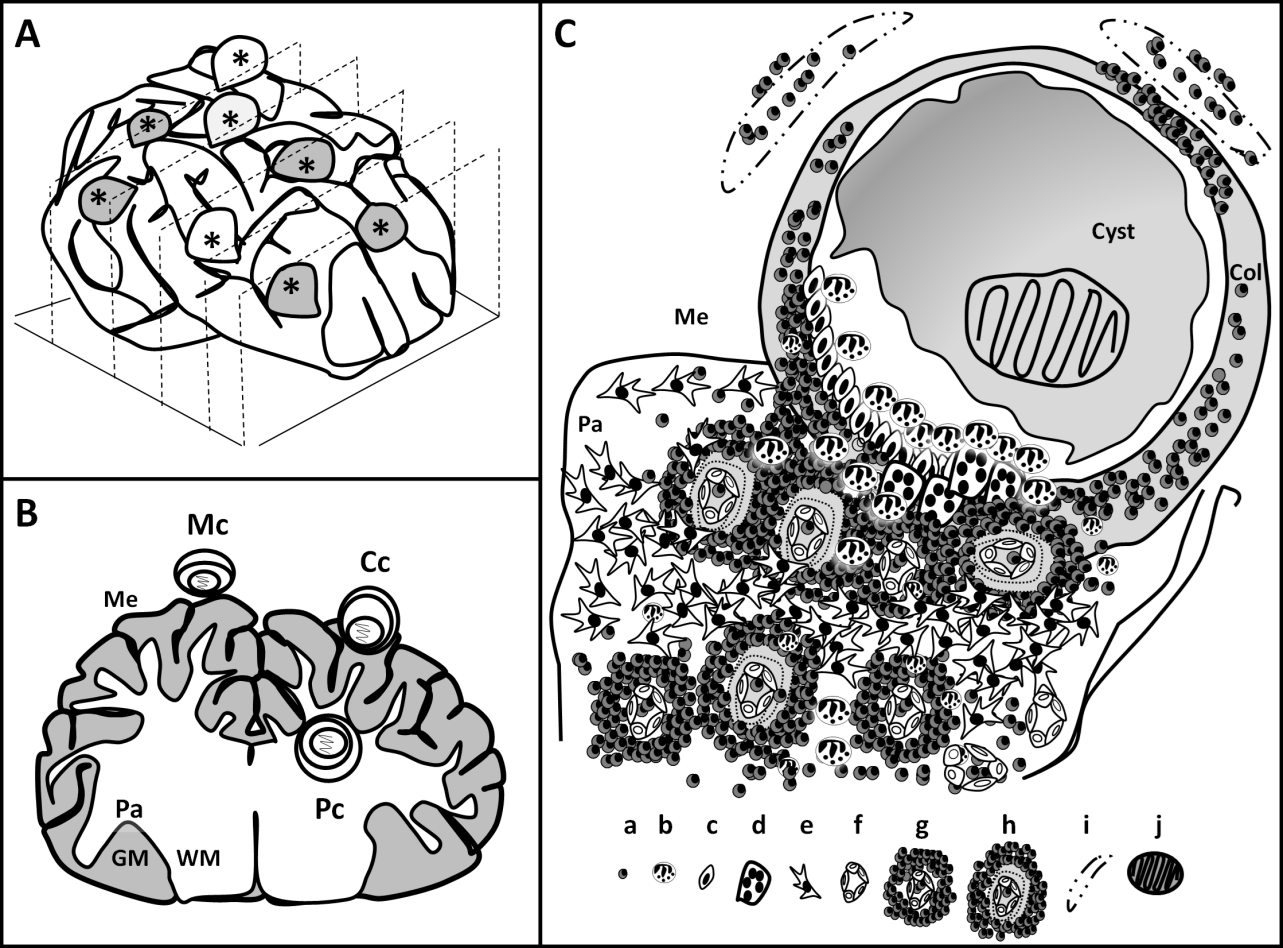
Cysticercus location. (A) Shows the orientation of the coronal sections used in this study. *: cysticercus; gray and white represent blue stained and clear capsules, respectively. (B) Coronal brain section showing the different cysticerci locations: Mc: Meningeal cysticercus, Cc: Corticomeningeal cysticercus and Pc: Parenchymal cysticercus. Me: Meninges. Pa: Parenchyma. WM: White matter. GM: Gray matter. **(**C) Corticomeningeal inflammatory response in parenchymal and meningeal tissue. a: lymphocyte, b: eosinophil, c: ephitelioid cell, d: giant multinucleated cell, e: astrocyte, f: healthy vessels, g: inflammated parenchymal vessel, h: inflammated parenchymal vessel with thick collagen layer, i: meningeal vessel, j: scolex.

Brains previously fixed in 10% neutral buffered formalin for 24 hours were cut in coronal 10–12 mm slices before collecting samples (Fig 1A). Biopsies with the parasite and adjacent brain tissue were taken, fixed in buffered formalin and embedded in paraffin. Serial 4-μm thick sections, cut following the same coronal orientation of the brain slices, were mounted on slides for histological analysis by hematoxylin–eosin (HE) and Masson’s Trichrome stains. Only complete cysticercus sections (including cysticercus wall and scolex) and surrounding inflammatory reaction were considered. Cysticerci that were no longer structurally recognizable, as well as degenerated cysticerci and cicatricial lesions were excluded [23]. Each cysticercus was represented by one section as explained below.

### Image acquisition

Microphotographs were taken with 40X magnification with a Carl Zeiss microscope with AxioVision software and each image was saved in the Joint Photographic Experts Group (.jpg) format. Individual 40X images of the studied slides were obtained by capturing images with a 10 to 20% overlap between contiguous fields; the software stitched the individual.jpg images together to form a single large image (“cysticercus map”).

### Cysticercus contact to brain tissue

Each cysticercus was classified into one of three location categories: parenchymal, meningeal, or corticomeningeal. Parenchymal cysticerci were totally surrounded by parenchymal tissue and meningeal cysticerci by meninges (Fig 1B). Corticomeningeal cysticerci had a parenchymal and a meningeal region (Fig 1B). The coronal orientation of the sections (Fig 1A) made any involvement with parenchymal tissue evident and thus the location of the cysticercus could not be mistaken; this allowed evaluation of only one section per cysticercus, mainly around its center. The variable CP (“contact with parenchyma”) refers to the proportion of the cysticercus in contact with parenchymal tissue relative to the total perimeter.

### Inflammatory response

The immune infiltrate in each cysticercus was classified into four separate inflammatory stages (IS), IS1 to IS4 as previously described [27]. In IS1 (low inflammation) the parasites were surrounded only by a thin layer of collagen (fibrosis), in IS2 (moderate inflammation) there was a sparse inflammatory infiltrate in between the collagen fibers; in IS3 (strong inflammation), granuloma formation was evident (cellular infiltrate in layers, together with the fibrosis) and in IS4 (severe inflammation) the parasite was surrounded by an eosinophil-rich exuberant infiltrate with fibrosis and obvious degeneration of the vesicular membrane [24,25,26]. The inflammatory score composite (ISC) was calculated by determining the percentage of the perimeter of the cysticercus for each IS, multiplied by the numerical value of the IS (1 to 4). The total ISC for one cysticercus was the sum of all IS components, as per the formula [27]:

ISC=(IS1%x1)+(IS2%x2)+(IS3%x3)+(IS4%x4);maximum=400. (1)

### Cysticercal damage

The methods detailing analysis of cysticercal damage were described previously [27]. The loss of identifiable structures at the cysticercus wall surface, tegument or subtegument layers was considered damage. The length in mm of the cysticercus wall that showed any degree of damage was measured using the AxioVision software and the percentage of this damage relative to the total perimeter of the cysticercus was expressed without units. [27,28]

### Disruption of the blood-brain barrier (BBB)

Pigs were injected with the intravital stain Evans blue two hours before being euthanized as previously described [23]; extravasation of the dye into the host tissue around the cysticercus (capsule) is a measure of the presence and degree of disruption of the BBB [29,30]. Clear and blue staining of the inflammatory reaction around the cysticercus represented intact and disrupted BBB, respectively [23].

### Data analysis

Inflammatory score composite (ISC) and cysticercal damage were continuous parameters; BBB disruption was treated as a dichotomic variable, and treatment groups were also categories. Associations according to cysticercal location were evaluated using the Mann-Whitney or Kruskal-Wallis tests (inflammation or cysticercal damage and treatment) and the Fisher’s exact test (treatment group and BBB disruption). A Gaussian family generalized linear model (GLM) with an identity link was used for multivariate analysis of corticomeningeal cysticerci to model the association between ISC and contact with the parenchyma (CP), adjusted by treatment group and the interaction between CP and treatment, considering each animal as a cluster. This final model was defined after testing each covariate (BBB disruption, cysticercal damage, treatment and CP) in a sequence of nested models with likelihood test. All statistical analyses were performed with a 95% significance level using the statistical software STATA (STATA Corp LP, College Station, TX) 11.0.

## Results

### Data set

The number of cysticerci in each pig brain was variable. The range was 3–95 considering all cysticerci (371 before selection of samples) in all groups; total parasites per pig in each group were as follows: D0 (8, 14 and 88), D2 (11, 27, 51 and 95) and D5 (4, 6, 10 and 57). Pigs in the D2 group had greater number of cysticerci than untreated pigs (D0) and the D5 group (total of 102 versus 56 and 38, respectively). Parenchymal cysticerci were more frequent: 105 out of 196 total cysticerci (53.6%) compared to 38 (19.4%) meningeal cysticerci and 53 (27.0%) corticomeningeal cysticerci (Table 1).

**Table 1:** Cysticercal location according to treatment group.

	D0 (N = 3 pigs)	D2 (N = 4 pigs)	D5 (N = 4 pigs)	Total
**Parenchymal cysticerci**	30	48	27	105 (53.6%)
**Corticomeningeal cysticerci**	11	37	5	53 (27.0%)
**Meningeal cysticerci**	15	17	6	38 (19.4%)
**Total**	56 (28.6%)	102 (52.0%)	38 (19.4%)	196 (100%)

D0, D2, D5: Treatment groups.

### Inflammation (ISC) and cysticercal damage in untreated cysticerci according to location

Inflammatory stages (IS1-IS4) and morphology varied according to the cysticercus location (total or partial parenchyma or meninges). Parenchymal IS1 had a collagen layer of variable thickness, whereas in meninges this layer was typically very thin; inflammatory cells were absent in both kinds of tissue. IS2 represented an increase in the collagen layer and the appearance of scattered cells both for parenchyma and meninges. Areas of IS3 could be found in untreated pigs (D0), although these were focal and restricted to a very small fraction of the cysticercus perimeter (not enough to increase the overall ISC value, thus not leading to statistical differences between locations in this group (Table 2).

**Table 2:** ISC and cysticercal damage characteristics by location groups according to treatment.

	Parenchymal (P) Median (n, Range)	Corticomeningeal (C) Median (n, Range)	Meningeal (M) Median (n, Range)	p-value^a^		
				P vs. C	P vs. M	C vs. M
**ISC**						
D0	210.5 (30, 113–400)	200 (11, 156–245)	187 (15, 100–237)	0.702	0.243	0.132
D2	229 (48, 100–400)	200 (37, 100–268)	190 (17, 106–255)	0.003	0.002	0.520
D5	300 (27, 154–400)	265 (5, 147–300)	190 (6, 104–300)	0.010	0.004	0.350
**Cysticercal damage**						
D0	0 (30, 0–100)	0 (11, 0–28)	0 (15, 0–25)	0.955	0.523	0.504
D2	0 (48, 0–100)	0 (37, 0–16)	0 (17, 0–77)	<0.001	0.103	0.185
D5	69 (27, 0–100)	32 (5, 13–100)	24 (6, 6–100)	0.528	0.659	0.454

n: number of cysticerci.

ISC: Inflammatory score composite (see Methods).

^a^ Mann-Whitney’s test.

**Fig 2:**
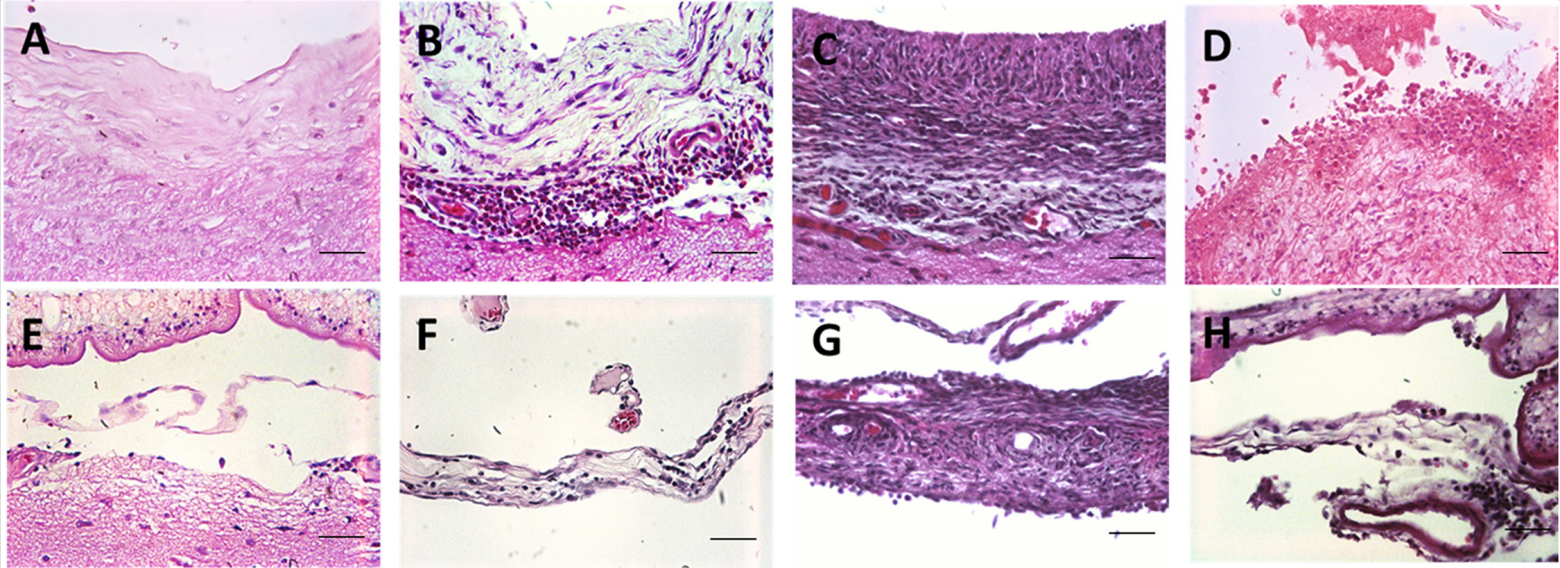
Inflammatory stages in parenchymal and meningeal cysticerci. (A), (B), (C) and (D) are IS1, IS2, IS3 and IS4 in parenchymal tissue, respectively. Collagen and number of cells tend to increase with the degree of inflammation, as previously described by Alvarez [24]. (E), (F) and (G) are examples of IS1, IS2 and IS3 in meningeal tissue, respectively. The collagenous areas in meningeal tissue are usually thinner than in parenchymal tissue and the number of inflammatory cells are decreased compared to the parenchyma tissue. (H) Shows inflamed meningeal vessels—note the inflammatory cells surrounding the vessels (All pictures are from Haematoxylin and eosin stain slides: 400X; bar: 50 μm).

Some differences between parenchymal and meningeal reactions could be seen on IS3; fibrosis, multinucleated cells and inflamed vessels (i.e., surrounded by inflamed cells, mostly eosinophils and lymphocytes) were more abundant in parenchyma than in meninges, and astrogliosis was, by nature of their location, exclusive to parenchyma (Fig 2). In corticomeningeal cysticerci, each distinct region (parenchymal and meningeal) behaved as either parenchymal or meningeal tissue. Inflammatory stages were distributed similarly between both regions.

### Effect of treatment on inflammation (ISC) and cysticercal damage according to location

ISC in D0, D2 and D5 was compared according to cysticerci location using the Mann-Whitney test. In untreated brains (D0), median values of ISC were not statistically different between parenchymal, meningeal or corticomeningeal cysticerci, indicating that inflammatory stages (IS1 to IS4) were distributed similarly among the three types of cysticerci. On the contrary, in D2 and D5 parenchymal cysticerci were statistically different from corticomeningeal (p = 0.03 and 0.01, respectively) and meningeal cysticerci (p = 0.002 and 0.004, respectively; Table 2). Treatment also resulted in higher ISC values after 2 and 5 days in parenchymal cysticerci than in corticomeningeal and meningeal cysticerci. (ISC up to 400, which corresponded to larger extensions of the higher stages of inflammation). These changes were statistically significant for parenchymal cysticerci (p<0.001, Kruskall-Wallis test) but not for corticomeningeal (p = 0.206) nor meningeal cysticerci (p = 0.898; Table 2).

There was a trend towards more cysticercal damage 5 days after treatment in the three locations (Table 2). Comparing cysticerci locations, only at D2 parenchymal cysticerci showed significantly more damage than corticomeningeal cysticerci (p<0.001).

**Fig 3:**
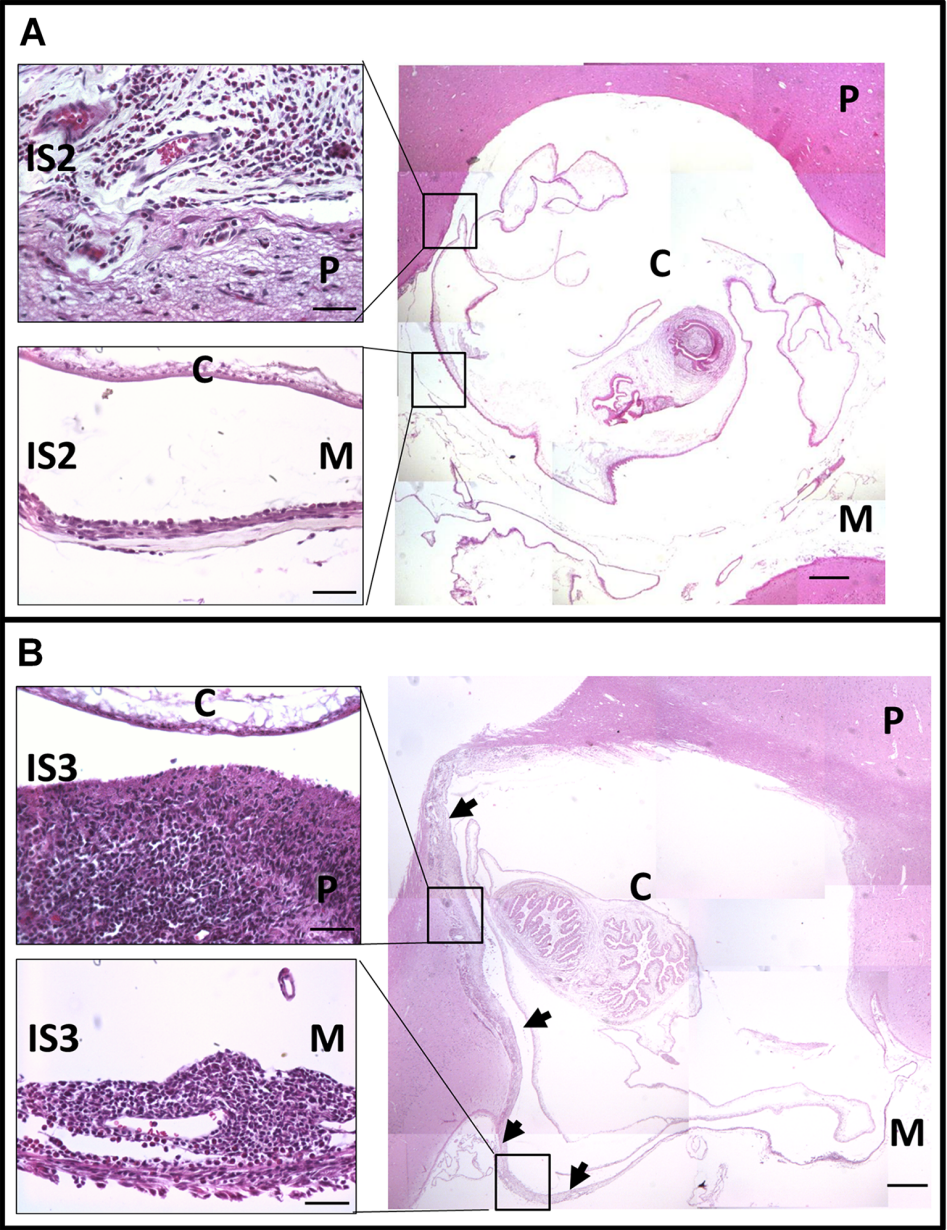
Histological changes in corticomeningeal cysticerci before and after treatment. (A) Untreated (D0) corticomeningeal cysticercus. The figure shows IS2 in both parenchyma and meninges region. IS3 is absent from both regions. (B) Treated (D5) corticomeningeal cysticercus. Both regions present IS3, which has a larger extension in the parenchymal region than in the meningeal region. *P*: Parenchymal region. *M*: Meningeal region. *C*: Cysticercus. *Arrows*: Inflammatory stage grade 3 (IS3) limits. (All figures are from Haematoxylin and eosin stain slides. Main figures, right: 40X; bar: 500 μm. Amplified panels, left: 400X; bar: 50 μm).

After treatment, the main histological changes were seen in parenchymal tissue: IS3 was clearly more frequent and greater in D2 and D5 cysticerci than in D0. Also, astrocytic gliosis associated to IS3 and IS4 was more intense in the treated groups than in the untreated but only in the parenchyma. While IS3 could be seen in meningeal tissue, it was considerably less frequent and not as widespread as in parenchyma, and only a few of the treated cysticerci showed an increase (Fig 2). In corticomeningeal cysticerci, inflammatory stages were distributed similarly between parenchymal and meningeal regions. Following treatment, the extent of higher grade IS3 inflammation around the cysticercus increased in parenchymal cysticerci but was only rarely seen in meningeal cysticerci, resulting in higher ISC values for parenchymal cysticerci (Fig 3A and 3B).

### Treatment affected BBB disruption only in parenchymal cysticerci

Anthelmintic treatment has been shown to increase the permeability of the BBB in pericystic tissue and this is related to inflammation [23]. This effect was observed in parenchymal cysticerci; the proportion of cysticerci showing disrupted BBB (blue staining) increased from 67% in the untreated group to 85% in D2 and 96% in D5.

To evaluate the relationship between treatment and the increase of the disruption of the BBB in meningeal, parenchymal and corticomeningeal cysticerci separately, we used the Fisher’s exact test. Only parenchymal cysticerci showed a statistically significant association between treatment and BBB disruption (p = 0.013; Fisher’s exact test). In corticomeningeal and meningeal cysticerci there were no apparent associations between treatment (D2 and D5) and an increased BBB disruption (Table 3).

**Table 3:** Effect of cysticercal location on the association of treatment and disruption of the BBB.

	D0 blue/total (%)	D2 blue/total (%)	D5 blue/total (%)	p-value^a^
**Parenchymal**	20/30 (67%)	41/48 (85%)	26/27 (96%)	0.013
**Corticomeningeal**	9/11 (81%)	30/37 (82%)	4/5 (80%)	1.000
**Meningeal**	10/15 (67%)	13/17 (76%)	4/6 (67%)	0.802

^a^ Fisher’s exact test.

### Inflammation in corticomeningeal cysticerci was proportional to the extent of contact with parenchyma

Corticomeningeal cysticerci were used to confirm that the inflammatory effect of treatment occurred mainly or only on parenchymal tissue. After determining that these cysticerci had comparable values of CP at D0, D2 and D5, ISC was calculated separately for the parenchymal and meningeal regions (Table 4).

**Table 4:** Inflammation in corticomeningeal cysticerci (C) according to treatment and contact with parenchyma or meninges.

C (n = 53)	D0	D2	D5	p-value^a^		
	Median (range)	Median (range)	Median (range)	D0 vs D2	D0 vs D5	D2 vs D5
**Contact with parenchyma (CP)**	44 (29–90)	50 (10–94)	40 (37–67)			
**ISC in parenchyma**	205 (152–275)	200 (100–279)	275 (122–300)	0.350	0.098	0.047
**ISC in meninges**	200 (100–245)	200 (100–300)	300 (100–300)	0.951	0.255	0.168

n: number of cysticerci.

^a^ Mann-Whitney’s test.

A bivariate analysis comparing ISC of these cysticerci according to treatment, measured separately for parenchyma and meninges, showed that inflammation in the meningeal region did not follow any discernible pattern of change after treatment, while inflammation in the parenchymal region increased and correlated to the proportion of the cysticercus in contact with the brain parenchyma. In a stratified analysis of ISC, using three separate GLM, D5 had the highest regression coefficient for CP, and with a significant p = 0.049, which indicates that inflammation at this time point of the treatment was significantly higher in cysticerci with more contact with parenchyma (Table 5).

**Table 5:** Stratified analysis for ISC for corticomeningeal cysticerci.

ISC	RC	CI (95%)	p-value^a^
**CP in D0 (n = 11)**	-0.900	-1.836 to 0.037	0.060
**CP in D2 (n = 37)**	0.050	-0.70 to 0.797	0.898
**CP in D5 (n = 5)**	1.803	-0.028 to -3.634	0.049

n: number of cysticerci.

CP: Contact with parenchyma.

RC: Regression coefficient.

CI: Confidence interval.

^a^ Generalized Linear Model.

## Discussion

The clinical expression and response to treatment in human NCC vary greatly according to whether the parasitic larvae are located in the brain parenchyma or subarachnoid space [3]. In human NCC parenchymal cysticerci are associated with seizures and respond better to antiparasitic treatment, whereas subarachnoid cysticerci commonly cause hydrocephalus and intracranial hypertension and their response to antiparasitic therapy is poor [5]. In order to study the effect of the location of cysticerci in the inflammation after treatment in pigs, we used histological specimens from naturally infected pigs to investigate the characteristics of inflammatory reaction around *T*. *solium* cysticerci according to their location in the brain parenchyma, meninges, or in both locations, and the effect of anthelmintic treatment on the above variables. The main finding is that exacerbation of pericystic inflammation on day 5 after praziquantel treatment is location-dependent: Inflammation was proportional to the extent of contact of the cysticercus with the brain parenchyma (Fig 1C). The relative lack of host response in the subarachnoid tissue following treatment could help explain the clinical observation that extraparenchymal cysticerci are commonly refractory to treatment.

Other studies in humans and pigs with NCC previously demonstrated that treatment with anthelmintic drugs increases inflammation in the brain parenchyma and cysticercal damage [11,15]. Our data demonstrate that without treatment this inflammatory response is similar between parenchymal, meningeal and corticomeningeal cysticerci, but significantly increases following treatment (day 2 and day 5) in parenchymal cysticerci compared with those in meningeal and corticomeningeal locations (Table 2).

Enhanced inflammatory responses in the parenchyma compared to the meninges may be due to differences in the vascular network within the tissues [18,21]. Parenchymal tissue has denser vascularization and more microvascularization, which would be expected to result in an increase in the number of inflamed vessels as well as a greater number of infiltrating cells in the parenchymal side of corticomenigeal cysticerci. For the same reason, fewer drugs, and in lesser amounts, may reach the meninges compared to the parenchyma, resulting in the observed unresponsiveness of extraparenchymal NCC.

Treatment was associated with BBB disruption only in parenchymal cysticerci. The proportion of Evans blue staining increased from 67% on D0 to 85% and 96% in D2 and D5, respectively, while there was no similar increase in the other locations (Table 3). Differences in BBB damage may also be explained by differences in the vasculature network as mentioned above, as well as differences in the junctional structure within vessels.

Antiparasitic treatment of human parenchymal NCC is frequently associated with an exacerbation of neurological symptoms, particularly seizures, which peak between the second and the fifth day after treatment onset [31]. Also, the increase in treatment related symptoms is frequently accompanied with the appearance of or increase in enhancement as seen using MRI imaging. These early side effects are very likely due to the induction of pericystic inflammation in parenchymal cysticerci. Likewise, in this report as well as in earlier studies we saw an exacerbation of inflammation and disruption of the BBB of parenchymal cysticerci during the same time frame as seen in humans. As for clinical manifestations in the animals during the five days of treatment, convulsions have so far not been reported nor were observed this time; evident signs of discomfort were also absent and it is not known if they would have been seen under constant observation.

The present study has certain limitations. Inflammation, location and damage to the parasite were evaluated using newly constructed score variables. These included the inflammatory score composite (ISC), based upon descriptions by Alvarez [24]; cysticercal damage, used previously by our group [27, 28] and degree of contact with brain parenchyma (CP), described in the present work. While we worked with small numbers of animals and could not properly account for intra-group correlation (cysticerci collected from a same pig can have similar features, which can create an intra-group correlation), we used robust standard errors and analyzed each pig as a cluster. Despite these limitations, our findings suggest a significant association between the degree of contact with the parenchyma and treatment induced inflammation. The enhanced inflammation in the parenchyma may in part also be due to decreased concentration of active drug in the meninges compared to the parenchyma. A major difference between the two locations is the quantity and unique character of the blood vessels. Because of the increased quantity of microvessels in the parenchyma, it may be that the concentration of cysticidal drug may be increased around parenchymally located cysticerci compared to those in the meninges. The consequence would be an increase in cysticercal damage and antigen release [32] inducing a greater degree of inflammation.

In summary, there are clear differences between the immune response profiles according to the location of each cysticercus in treated and untreated pigs. These differences likely contribute to known differences in treatment efficacy between parenchymal cysticerci and subarachnoid NCC. More thorough knowledge of the factors causing this differential response, including whether PZQ pharmacodynamics act differentially in pigs and in humans, should contribute to the understanding of NCC pathogenesis and its better management.
